# Mining Pure, Strict Epistatic Interactions from High-Dimensional Datasets: Ameliorating the Curse of Dimensionality

**DOI:** 10.1371/journal.pone.0046771

**Published:** 2012-10-12

**Authors:** Xia Jiang, Richard E. Neapolitan

**Affiliations:** 1 Department of Biomedical Informatics, University of Pittsburgh, Pittsburgh, Pennsylvania, United States of America; 2 Division of Health and Biomedical Informatics, Department of Preventive Medicine, Northwestern University Feinberg School of Medicine, Chicago, Illinois, United States of America; Cleveland Clinic Lerner Research Institute, United States of America

## Abstract

**Background:**

The interaction between loci to affect phenotype is called *epistasis*. It is strict *epistasis* if no proper subset of the interacting loci exhibits a marginal effect. For many diseases, it is likely that unknown epistatic interactions affect disease susceptibility. A difficulty when mining epistatic interactions from high-dimensional datasets concerns the *curse of dimensionality*. There are too many combinations of SNPs to perform an exhaustive search. A method that could locate strict epistasis without an exhaustive search can be considered the *brass ring* of methods for analyzing high-dimensional datasets.

**Methodology/Findings:**

A *SNP pattern* is a Bayesian network representing SNP-disease relationships. The *Bayesian score* for a SNP pattern is the probability of the data given the pattern, and has been used to learn SNP patterns. We identified a bound for the score of a SNP pattern. The bound provides an upper limit on the Bayesian score of any pattern that could be obtained by expanding a given pattern. We felt that the bound might enable the data to say something about the promise of expanding a 1-SNP pattern even when there are no marginal effects. We tested the bound using simulated datasets and semi-synthetic high-dimensional datasets obtained from GWAS datasets. We found that the bound was able to dramatically reduce the search time for strict epistasis. Using an Alzheimer's dataset, we showed that it is possible to discover an interaction involving the APOE gene based on its score because of its large marginal effect, but that the bound is most effective at discovering interactions without marginal effects.

**Conclusions/Significance:**

We conclude that the bound appears to ameliorate the curse of dimensionality in high-dimensional datasets. This is a very consequential result and could be pivotal in our efforts to reveal the dark matter of genetic disease risk from high-dimensional datasets.

## Introduction

In Mendelian diseases, a genetic variant at a single locus may give rise to a disease [1]. However, in many common diseases, it is likely that manifestation of the disease is due to genetic variants at multiple loci, with each locus conferring modest risk of developing the disease. For example, there is evidence that gene-gene interactions may play an important role in the genetic basis of hypertension [2], sporadic breast cancer [3], and other common diseases [4]. The interaction between two or more genes to affect a phenotype such as disease susceptibility is called *epistasis*. Biologically, epistasis likely arises from physical interactions occurring at the molecular level. Statistically, epistasis refers to an interaction between multiple loci such that the net effect on phenotype cannot be predicted by simply combining the effects of the individual loci. Often, the individual loci exhibit weak marginal effects; sometimes they may exhibit none. We say it is a *pure epistatic* interaction if no single locus exhibits a marginal effect, and we say it is a *strict epistatic* interaction if no subset of the interacting loci exhibits a marginal effect.

The ability to identify epistasis is important in understanding the inheritance of many common diseases. For example, studying genetic interactions in cancer is essential to further our understanding of cancer mechanisms at the genetic level. Many cancer-associated mutations and interactions among the mutated loci remain unknown. For example, highly penetrant cancer susceptibility genes, such as BRCA1 and BRCA2, are linked to breast cancer [5]. However, only about 5 to 10 percent of breast cancers can be explained by germ-line mutations in these single genes. Most women with a family history of breast cancer do not carry germ-line mutations in the single highly penetrant cancer susceptibility genes, yet familial clusters continue to appear with each new generation [6]. This kind of phenomenon is not yet well understood, and undiscovered mutations or undiscovered interactions among mutations are likely responsible.

The most common genetic variation is the *single nucleotide polymorphism* (*SNP*) that results when a nucleotide that is typically present at a specific location on the genomic sequence is replaced by another nucleotide. In most cases a SNP is biallelic, that is it has only two possible values among A, G, C, and T (the four DNA nucleotide bases). The less frequent (rare) allele must be present in 1% or more of the population for a site to qualify as a SNP [7]. The human genome is estimated to contain 15–20 million SNPs. In what follows we will refer to SNPs as the loci investigated when searching for a correlation of some loci with a phenotype such as disease susceptibility.

The advent of high-throughput technologies has enabled *genome-wide association studies* (*GWAS*). A GWAS involves genotyping representative SNPs in individuals sampled from a population. A GWAS dataset can concern millions of SNPs and may soon concern billions. Data in which each record has such a large number of attributes is called *high-dimensional*. In case-control GWAS we identify the disease status along with the values of the SNPs. Such studies provide researchers unprecedented opportunities to investigate the complex genetic basis of diseases. By looking at single-locus associations, researchers have identified over 150 risk loci associated with 60 common diseases and traits [8], [9], [10], [11], [12], [13].

However, single-SNP investigations could not detect complex epistatic interactions in which each locus by itself exhibits little or no marginal effect. To fully exploit genomic data and possibly reveal a great deal of the dark matter of genetic risk, it is critical that we analyze such data using multi-locus methods, which investigate 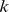-SNP patterns. Briefly, a 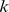-*SNP pattern* is a Bayesian network (BN) model that describes the relationship between 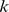 SNPs and disease status, and is described in detail in the Methods Section.

When investigating SNP patterns, in some way we must score the patterns to determine which patterns are most noteworthy. Standard techniques such as linear regression may not work well because both the predictors and the target are discrete. One well-known technique is *Multifactor Dimensionality Reduction* (*MDR*) [14]. MDR combines two or more variables into a single variable (hence leading to dimensionality reduction); this changes the representation space of the data and facilitates the detection of nonlinear interactions among the variables. MDR has been successfully applied to detect epistatic interactions in diseases such as sporadic breast cancer [3] and type II diabetes [15]. Using 28,000 simulated datasets and a real Alzheimer's GWAS dataset, Jiang et al. [16] evaluated the performance of 22 BN scoring criteria and MDR when scoring SNP patterns. They found that several of the BN scoring criteria performed substantially better than other scores and MDR. The BN scores that performed best were ones that computed the *Bayesian score*, which is the probability of the data given the pattern.

A difficulty when learning SNP patterns from high-dimensional GWAS datasets concerns the *curse of dimensionality*. For example, if we only investigated all 0, 1, 2, 3 and 4-SNP patterns when there are 500,000 SNPs, we would need to investigate 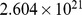 patterns. Therefore, researchers have worked on developing heuristic search methods that investigate multiple loci using a GWAS dataset. Lasso, which is a shrinkage and selection method for linear regression [17], [18], was applied to this task [19], [20]. However, linear regression obviously has difficulty handling nonlinear epistatic interactions. Other methods include permutation testing [21], [22], the use of ReliefF [23], [24], random forests [25], predictive rule inference [26], a variational Bayes algorithm [27], a Bayesian marker partition method [28], an MCMC approximate model averaging technique [29], a Markov blanket method [30], the use of maximum entropy [31], the use of Bayesian networks and greedy search [32], and an ensemble-based method that uses boosting [33].

Each of these methods has at least one of the following shortcomings: 1) It only investigates two-locus interactions and still requires quadratic time; or 2) It has only been shown to detect interactions in which only one interacting locus has no significant marginal effect. Many of the methods proceed in stages, using the first stage to identify promising SNPs, which in some way are investigated further in the second stage. Strict epistasis constitutes the worst-case in terms of detecting disease associations because such associations are only observable if all interacting SNPs are included in the disease model. None of these two-stage methods made any progress towards detecting strict epistasis. So, Evans et al. [34] conclude that “it is preferable to perform an exhaustive two-locus search across the genome rather than either of the two-stage procedures that we examined. Otherwise, investigators risk discarding significant loci that only exhibit small effects at the margins.”

An exhaustive search is not possible when there are millions of SNPs. So some researchers turned their efforts to reducing the search space based on ancillary knowledge. You et al. [35] performed a two-stage application of MDR. The first stage is a within-gene search in which all combinations of SNPs allocated to the same gene are investigated. Briggs et al. [36] identified promising regions harboring epistatic candidates by looking for concordance in affected sibling pairs. Jiang et al. [37] investigated all 2-loci combinations where one of the loci was previously known to be associated with the disease. Perhaps the most promising technique for reducing the search space is to restrict the search space for candidate gene sets by using knowledge about molecular pathways [38].

However, once the search space is reduced, we can still be left with a large number of SNPs, prohibiting an exhaustive search of even the pruned dataset. Furthermore, in an agnostic study we are searching for possible interactions for which we have no previous knowledge. Therefore, a multi-stage technique that can effectively locate strict epistatic interactions could still be considered the *brass ring* of methods for analyzing high-dimensional datasets.

Initially it might seem that it is not possible to successfully prune our search for strict epistatic 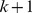-SNP interactions by investigating 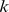-SNP patterns. This is likely the case when we are scoring the patterns. However, we have identified a bound (not a score) for SNP patterns. The bound provides an upper limit on the Bayesian score of any pattern that could be obtained by expanding a given pattern. For example, it gives an upper limit on the Bayesian score of any 2-SNP pattern that could be obtained from a particular 1-SNP pattern. We speculated that this bound might enable the data to tell us something about the promise of expanding a 1-SNP pattern even when there are no marginal effects. We tested the bound using 6000 simulated datasets developed from models of strict epistasis. We also injected epistatic interactions in two real GWAS datasets to create 2400 high-dimensional semi-synthetic datasets. In the case of both the simulated datasets and the semi-synthetics datasets, the bound was able to significantly reduce the search time for the epistatic interaction. This was the case regardless of the heritability used to generate the interaction and the dimension of the dataset. The average fraction of the search space investigated before finding the *true pattern* (the one representing the interjected interaction) was as little as 0.0004.

## Methods

We developed both simulated and semi-synthetic datasets based on models of strict epistasis. We compared the performance of the bound and Bayesian score in their ability to efficiently locate the true pattern. After providing background on Bayesian networks, we discuss specialized Bayesian networks called SNP patterns that represent relationships among SNPs and a disease. Then we show the algorithm used for the comparisons, and we describe the datasets we developed.

### Bayesian Networks

Let 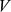 be a set of random variables, 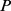 be a joint probability distribution of these random variables, and 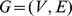 be a *directed acyclic graph* (*DAG*) where 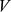 is the set of nodes in 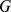 and 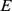 is the set of edges among these nodes. We say that 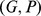 satisfies the *Markov condition* if for each variable 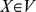, 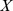 is conditionally independent of the set of all its nondescendents in 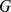 given the set of all its parents. If 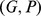 satisfies the Markov condition, we call 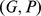 a *Bayesian network* (*BN*).

**Figure 1:**
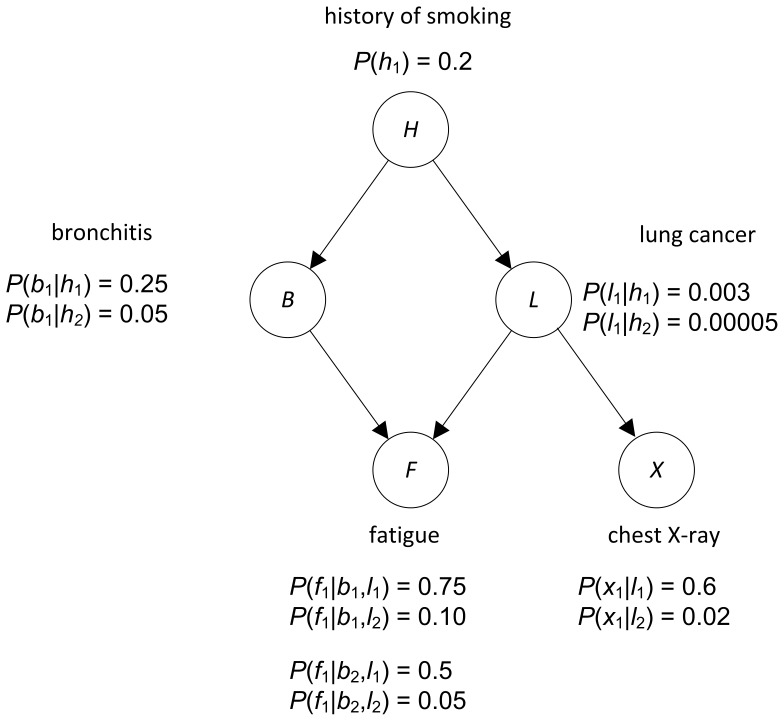
A BN for diagnosing lung disorders.

It is a theorem [39] that 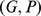 satisfies the Markov condition (and therefore is a BN) if and only if 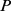 is equal to the product of its conditional distributions of all nodes given their parents in 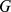, whenever these conditional distributions exist. That is, if our variables are 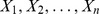, and 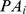 is the set of parents of 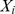, then
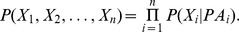
Due to the theorem just mentioned, a BN is often developed by first identifying a DAG that satisfies the Markov condition relative to our belief about the probability distribution of the nodes in the DAG, and then determining the conditional probability distributions for this DAG. Often the DAG is a causal DAG, which is a DAG in which there is an edge from 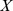 to 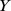 if and only if 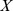 is a direct cause of 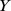 relative to the other nodes in the DAG. See [39] for a discussion as to why a causal DAG should often satisfy the Markov condition with the probability distribution of the variables in the DAG. Figure 1 shows a BN representing the causal relationships among variables related to lung disorders. In this network, 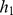 denotes an individual has a smoking history and 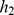 denotes that the individual does not. The other variables have similar denotations. Using this BN, we can determine conditional probabilities of interest using the BN and a BN inference algorithm. For example, if a given individual has a smoking history, a positive chest X-ray, and is fatigued, we can determine the conditional probability of the individual having lung cancer. That is, we can compute 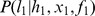. These inference algorithms exploit Bayes' Theorem and are efficient for a large class of BNs [39].

Methods have been develop both for learning the parameters in a BN and the structure (DAG) from data. Pierrier et al. [40] provide a recent review of the literature concerning BN structure learning. We review structure learning using the Bayesian score because that method is relevant to the research described here.

A *DAG model* consists of a DAG 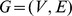 where 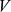 is a set of random variables, and a parameter set 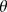 whose members determine conditional probability distributions for 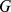, but without specific numerical assignments to the parameters. The task of learning a unique DAG model from data is called *model selection*. As an example, if we had data on a large number of individuals concerning lung disorders we might be able to learn the DAG in Figure 1 from data. When the edges represent causal influences, this means that we can learn causal influences from data.

In the constraint-based structure learning approach [41], we try to learn a DAG model from the conditional independencies that the data suggest are present in the generative probability distribution 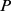. By the *generative probability distribution* 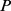 we mean the underlying joint probability distribution of the random variables, making the assumption that such a distribution exists. In the score-based structure learning approach [39], we assign a score to a DAG based on how well the DAG fits the data.

A straightforward score, called the *Bayesian score*, is the probability of the DAG model given the 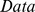, which is as follows:
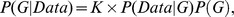
where 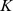 is a normalizing constant. If we assign a uniform prior probability distribution to the DAG models, we can score them using the likelihood 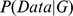, and that is the approach taken here. So we will simply refer to the likelihood as the score. For a DAG 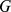 containing a set of discrete random variables 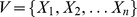 and 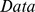 such that each data item is a vector of values of all random variables in 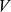, Cooper and Herskovits [42] develop the following formula for this likelihood:
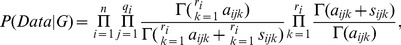
(1)where 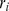 is the number of states of 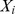, 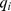 is the number of different instantiations of the parents of 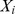, 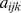 is the ascertained prior belief concerning the number of times 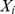 took its 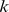
th value when the parents of 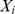 had their 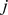
th instantiation, and 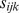 is the number of times in the data that 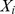 took its 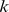
th value when the parents of 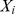 had their 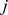
th instantiation.

The likelihood in Equation 1 assumes that our prior belief concerning each unknown parameter in each DAG model is represented by a Dirichlet distribution, where the hyperparameters 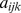 are the parameters for this distribution. Cooper and Herskovits [42] suggested setting the value of every hyperparameters 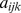 equal to 1, which amounts to assigning a prior uniform distribution to the value of each parameter (prior ignorance as to its value). They called this the *K2* score.

However, Heckerman et al. [43] showed that the K2 score does not assign the same score to Markov equivalent DAG models (two DAGs are *Markov equivalent* if they entail the same conditional independencies). For example, the DAGs 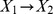 and 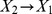 do not obtain the same score. They suggested determining the values of the hyperparameters from a single parameter 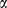 called the *prior equivalent sample size*. If we want to use a prior equivalent sample size and represent a prior uniform distribution for each variable (not parameter) in the network, for all 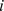, 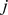, and 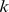 we set 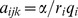 where 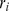 is the number of states of the 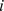
th variable and 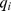 is the number of different instantiations of its parents. When we do this, the Bayesian score is called the *Bayesian Dirichlet equivalent uniform* (*BDeu*) score. That score is as follows:
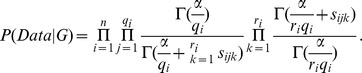
Heckerman et al. [43] showed that if we use a prior equivalent sample size, then Markov equivalent DAGs have the same score.

The Bayesian score does not explicitly include a DAG penalty. However, the penalty is implicitly determined by the hyperparameters 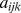. Silander et al. [44] show that if we use the BDeu score, then the DAG penalty decreases as 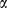 increases.

The Bayesian score decomposes into the product of local scores, one for each node 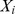 in the DAG. In the case of the BDeu score, the local score for 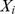 with parent set 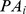 is given by
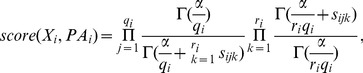
(2)where 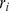 is the number of states of 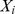, and 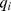 is the number of different instantiations of the nodes in 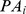. When learning a DAG model from data, we could try to maximize our local scores by looking for an optimal parent set 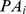 for each node 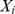. We could prune our search space if we had an upper bound on the local score for any parent set that could be obtained by adding parents to a current parent set 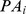. Singh and Moore [45] proved the following Theorem relevant to such a bound.

#### Theorem 1

*If in* *Equation* 2 *we fix the value of* 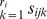 *for each* 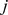 *, then* 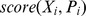 *is maximized if for each* 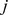 *,* 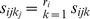 *for some* 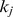 *. That is, for every combination of values of the parents of* 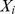 *, all data items that have those values have the same value of* 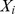.

This theorem could be used to obtain a bound on the local score that could be obtained by adding parents to a given parent set. However, the bound is a very loose bound (i.e. the bounds are much greater than the scores), and therefore the bound has not proven to be useful in pruning the search space.

### SNP Patterns and a Bound for SNP Patterns

**Figure 2:**
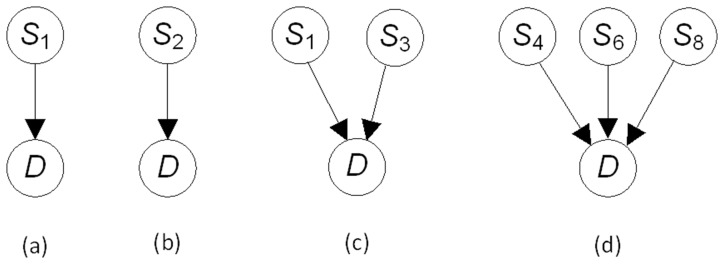
Four SNP patterns.

We could develop a DAG model that represents many factors that affect phenotype including inheritable allele variation, somatic mutations in alleles, environmental factors, and epigenetic phenomena such as DNA methylation. A subnetwork of that model contains only variables that represent inheritable allele variation (the variables whose values are obtained in a GWAS). If, for example, we have a 5-way interaction, two 3-way interactions, and two 2-way interactions, there are 15 SNPs in this subnetwork, all which have edges to the disease node 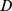. We have neither the data nor the computational time to score such networks. So we must settle for trying to learn pieces of the network, such as particular interactions, separately. These small subnetworks are the focus of this paper, and we call them *SNP patterns*. Examples of such patterns appear in Figure 2. The first two represent that a single SNP by itself is associated with the disease, the third one represents that two SNPs together are associated with the disease, and the fourth one represents that three SNPs together are associated with the disease. It is important to recognize that, for example, the pattern in Figure 2 (c) does not entail that 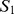 and 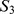 are interacting to affect 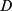. Each could be affecting it separately. Without making specialized mathematical assumptions, it is difficult to distinguish these two situations from data alone. Jiang et al. [37] provide one way to do this.

In the case of a SNP pattern 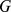 and 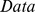 concerning the SNPs and disease in the pattern, the local BDeu score (Equation 2) is as follows:
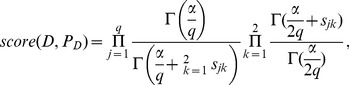
(3)where 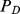 is the parent set of 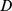 in the pattern, 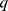 is the number of different instantiations of the parents of 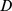, and 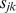 is the number of times in the data that 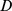 took its 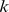
th value when the parents of 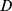 had their 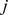
th instantiation.

Using Theorem 1, we can obtain a bound on the local score that could be obtained by adding parents to a given parent set. Given a SNP pattern and the score in Equation 3, an upper limit on the BDeu score of any SNP pattern obtained by adding more SNPs is as follows (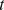 is the number of combined states of the SNPs added):
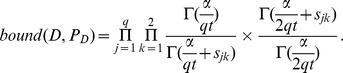
If all variables are binary, and we are looking only for a bound on adding one SNP, 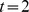, if it is a bound on adding two SNPs, 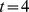, and if it is a bound on adding three SNPs, 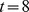.

As noted earlier, this is a very loose bound and would not be useful for provably pruning the search space. However, in the case of searching for strict epistatic interactions, perhaps it is asking too much to hope for provable results. We would achieve valuable progress if we could just often heuristically locate such interactions without an exhaustive search. We conjectured that the bound might enable us to do that, and that is what is investigated here.

### Algorithm

Suppose we have a dataset concerning SNPs and a disease, and our goal is to find a 2-SNP pattern with a particular BDeu score (Equation 3). This pattern could be the highest scoring pattern or it could be the true pattern representing an epistatic interaction used to generate the data. Often these two patterns are the same. We search for that pattern as follows. First, all the 1-SNP patterns are sorted by either their 2-SNP scores or their 2-SNP bounds. Let 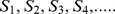 be the resultant sorted list of SNPs. We then investigate 2-SNP patterns according to the following order:
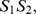


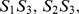


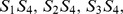


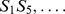
We stop when we locate the pattern for which we are searching, and we keep track of the number of patterns checked before we find that pattern.

This strategy extends readily to search for 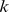-SNP patterns. The following is an algorithm for the general case:

**Algorithm 1** Find_Pattern


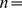
total number of SNPs;


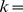
number of SNPs in the patterns we are investigating;


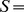
array containing the SNPs in sorted sequence;


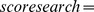
score of pattern we are trying to locate
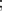


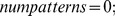


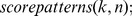
procedure 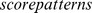
(int 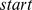, int 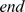);

if
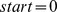



 

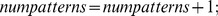



 

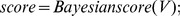



 if 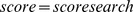


  output 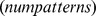;

  halt;

 endif

else

 for 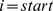 to 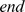


  

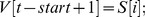



  

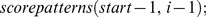



 endfor

endelse

Algorithm 1 constructs each candidate pattern in the array 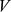. When 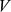 contains 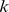 SNPs, the pattern is scored. If its score is equal to the score of the pattern for which we are searching, the algorithm outouts the number of patterns checked and halts. Note that if we are searching for a pattern representing a particular epistatic interaction, it will halt when we find any pattern with the same score as that pattern. However, in our experiments this unlikely event never occurred.

### Simulated Data Sets

Fisher et al. [46] created GAMETES, which is a software package for generating strict epistatic models with random architectures. We used GAMETES to develop 2-SNP, 3-SNP, and 4-SNP models of strict epistatic interaction. The software allows the user to specify the *heritability* and the *minor allele frequency* (*MAF*). We wanted to test a full range of MAFs (0.05 to 0.4) and relatively low and high heritabilities (0.05 and 0.2). However, we found that to obtain models with a low MAF, it was sometimes necessary to decrease the heritability. The actual MAFs and heritabilities used in the simulations are shown in Table 1.

**Table 1:** Properties of the strict epistatic models used in the simulated datasets.

# SNPs	MAF	Low Heritability	High Heritability
2	0.05	0.05	0.1
2	0.1	0.05	0.2
2	0.2	0.05	0.2
2	0.3	0.05	0.2
2	0.4	0.05	0.2
3	0.05	0.005	0.01
3	0.1	0.05	0.1
3	0.2	0.05	0.2
3	0.3	0.05	0.2
3	0.4	0.05	0.2
4	0.05	0.001	0.002
4	0.1	0.005	0.01
4	0.2	0.05	0.1
4	0.3	0.05	0.2
4	0.4	0.05	0.2

For each of the 30 combinations of MAF and heritability, we developed datasets in which there were 100 SNPs and 1000 SNPs. Each dataset had 1000 cases and 1000 controls. For each of the 60 variations, 100 datasets were generated, making a total of 6000 datasets. We used the BDeu score with 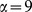 to score the SNP patterns and to compute their bounds using a given dataset. For each of the datasets, we first sorted the SNPs by their bounds. For the 2-SNP interactions, it was the bound on adding one SNP; for the 3-SNP interactions, it was the bound on adding two SNPs; and for the 4-SNP interactions, it was the bound on adding three SNPs. We then used Algorithm 1 to determine how many patterns were checked before the true pattern representing the interaction was discovered. In the case of the 1000 SNP datasets it was not computationally feasible to actually run Algorithm 1. Rather we simply determined the locations where the interacting SNPs appeared in the sorted list, and then using these locations we computed where Algorithm 1 would find the true pattern representing the interacting SNPs. We repeated this procedure with the SNPs sorted by their scores.

We did not vary the number of cases and controls because that number does not have a significant effect on the running time. The data needs to preprocessed to obtain the counts needed in the computation of the bound and the score. The only effect that the number of cases and controls has on the running time is that the pre-processing time increases linearly with the total number of cases and controls.

### Semi-Synthetic Datasets

Reiman et al. [13] developed a GWAS late onset Alzheimer's disease (LOAD) dataset on 312,317 SNPs from an Affymetrix 500 K chip, plus the measurement of a locus in the APOE gene, which is known to be predictive of LOAD. The dataset consists of 859 cases and 552 controls. See http://www.tgen.org/neurogenomics/data concerning this dataset. Hunter et al. [47] conducted a GWAS concerning 546,646 SNPs and breast cancer as part of the National Cancer Institute Cancer Genetic Markers of Susceptibility (CGEMS) Project. The dataset consists of 1145 cases and 1142 controls. See http://cgems.cancer.gov/ concerning this dataset.

We developed 2-SNP, 3-SNP, and 4-SNP models of strict epistatic interactions using GAMETES, and used the models to inject interacting SNPs into each of the real GWAS datasets resulting in semi-synthetic datasets. The models generated have the properties shown in Table 2. For each of the 12 models, 100 datasets were developed.

**Table 2:** Properties of the strict epistatic models used in the semi-synthetic datasets.

# SNPs	MAF	Heritability
2	0.05	0.1
2	0.1	0.2
2	0.15	0.3
2	0.2	0.4
3	0.05	0.01
3	0.1	0.04
3	0.15	0.1
3	0.2	0.2
4	0.05	0.001
4	0.1	0.01
4	0.15	0.04
4	0.2	0.1

We used the BDeu score with 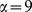 to score the SNP patterns and to compute their bounds using a given dataset. We used Algorithm 1 to search for the true pattern representing the interacting SNPs using both the bound and the score to sort the SNPs.

## Results

### Simulated Datasets

**Figure 3:**
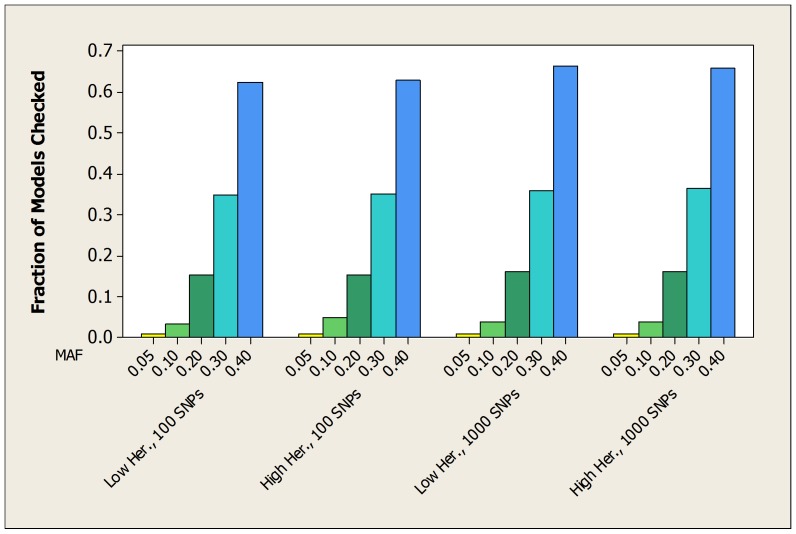
For the simulated datasets developed from 2-SNP models, the average fraction of the total number of patterns checked before the bound finds the true pattern.

**Figure 4:**
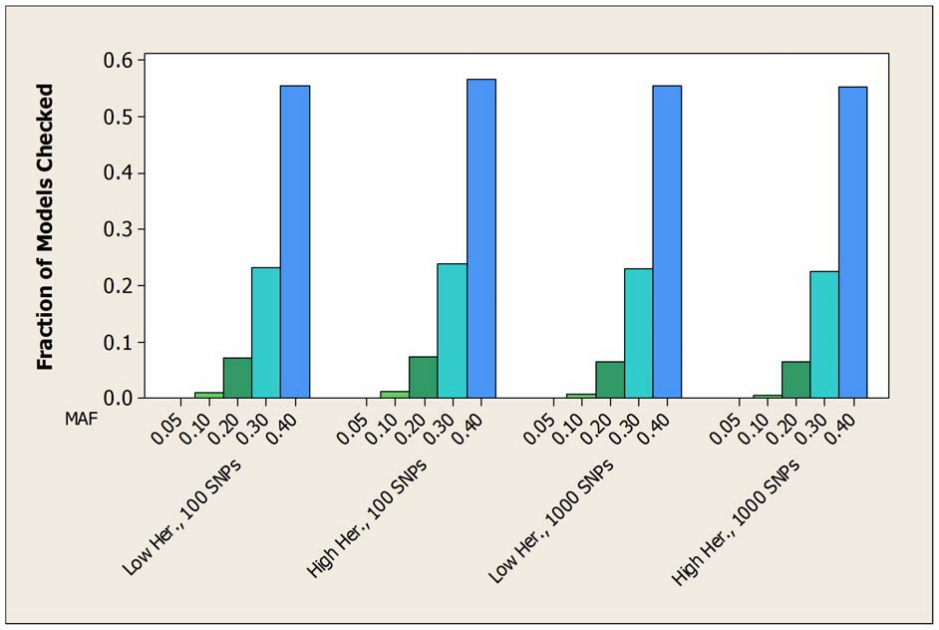
For the simulated datasets developed from 3-SNP models, the average fraction of the total number of patterns checked before the bound finds the true pattern.

**Figure 5:**
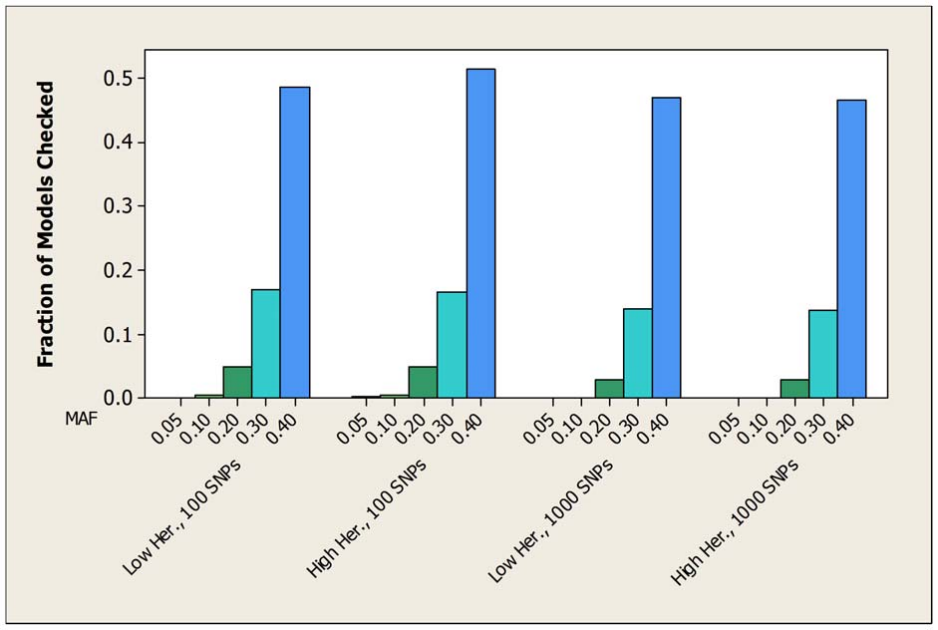
For the simulated datasets developed from 4-SNP models, the average fraction of the total number of patterns checked before the bound finds the true pattern.

For the simulated datasets developed from the 2-SNP models, Table 3 shows the average over all 100 datasets of the fraction of patterns checked by both the bound and the score before the true pattern is found. Tables 4 and 5 show the same information for the datasets developed from the 3-SNP and 4-SNP models. Figures 3, 4, and 5 show the average fraction of patterns checked by the bound in graphical format.

**Table 3:** For the simulated datasets developed from 2-SNP models, average fraction over all 100 datasets of the total number of patterns checked before finding the true pattern.

	100 Data Items				1000 Data Items			
	Low Heritability		High Heritability		Low Heritability		High Heritability	
MAF	Bound	Score	Bound	Score	Bound	Score	Bound	Score
0.05	0.007	0.042	0.007	0.042	0.008	0.044	0.008	0.048
0.1	0.033	0.133	0.047	0.134	0.037	0.148	0.037	0.139
0.2	0.151	0.356	0.153	0.385	0.159	0.386	0.159	0.404
0.3	0.348	0.594	0.349	0.554	0.359	0.571	0.362	0.555
0.4	0.623	0.641	0.629	0.696	0.662	0.673	0.656	0.714

**Table 4:** For the simulated datasets developed from 3-SNP models, average fraction over all 100 datasets of the total number of patterns checked before finding the true pattern.

	100 Data Items				1000 Data Items			
	Low Heritability		High Heritability		Low Heritability		High Heritability	
MAF	Bound	Score	Bound	Score	Bound	Score	Bound	Score
0.05	0.001	0.015	0.001	0.017	0.0005	0.006	0.0009	0.014
0.1	0.010	0.075	0.012	0.079	0.006	0.032	0.005	0.031
0.2	0.072	0.312	0.073	0.309	0.064	0.316	0.063	0.307
0.3	0.232	0.557	0.238	0.566	0.228	0.557	0.225	0.579
0.4	0.554	0.699	0.566	0.770	0.554	0.705	0.552	0.738

**Table 5:** For the simulated datasets developed from 4-SNP models, average fraction over all 100 datasets of the total number of patterns checked before finding the true pattern.

	100 Data Items				1000 Data Items			
	Low Heritability		High Heritability		Low Heritability		High Heritability	
MAF	Bound	Score	Bound	Score	Bound	Score	Bound	Score
0.05	0.001	0.007	0.002	0.012	0.0004	0.004	0.0005	0.015
0.1	0.005	0.052	0.005	0.053	0.001	0.036	0.001	0.037
0.2	0.048	0.271	0.048	0.275	0.028	0.262	0.028	0.283
0.3	0.170	0.554	0.165	0.544	0.139	0.534	0.137	0.552
0.4	0.486	0.674	0.514	0.751	0.471	0.751	0.466	0.713

These tables and figures show that the bound usually performs much better than we would expect by chance, and also performs substantially better than the score. When the MAF is small (0.05) the average fraction of patterns checked by the bound is at most around 0.008. By chance alone we would expect that average to be around 0.5. In general, the average fraction increases as the MAF increases. On the other hand, the heritability and the dimension of the dataset have little effect on the average fraction. It is somewhat surprising that the bound performs about as well when the heritability is low as when it is high. Since unknown genetic risk might confer low heritability, this is an encouraging result.

**Figure 6:**
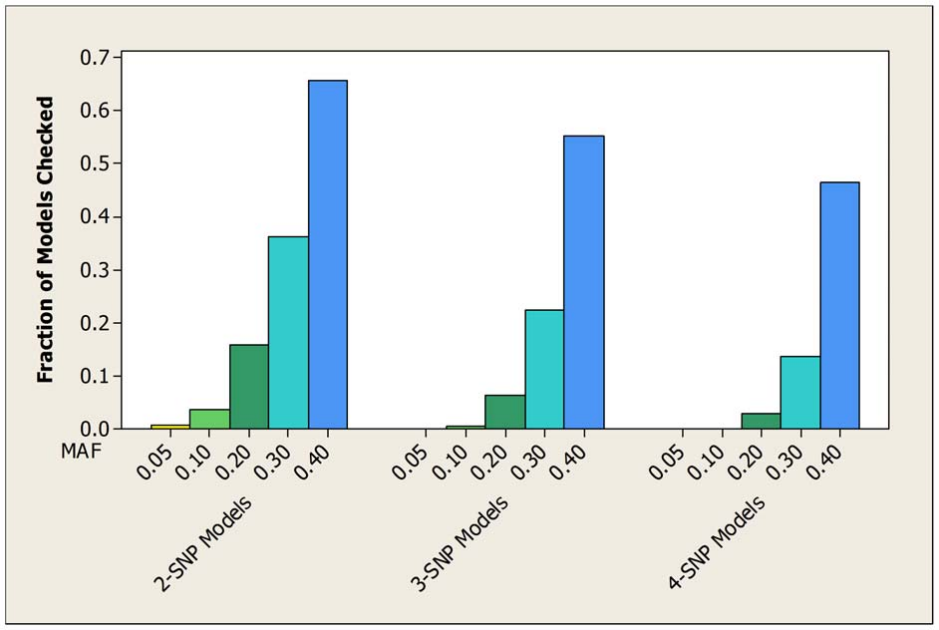
For the simulated datasets with 1000 SNPs and high heritability, the average fraction of the total number of patterns checked before the bound finds the true pattern.

The other variable that affects the performance of the bound is the number of SNPs involved in the epistatic interaction. That is, the performance improves as the number of SNPs increases. Figure 6 illustrates this using the experiments in which there were 1000 SNPs and the heritability was high.

In order to make these results more transparent, we did an analysis using actual times. As noted above, the performance does not seem to degrade as the dimension of the datasets increases (i.e., the results are about the same for the 100 SNP datasets and for the 1000 SNP datasets). If we assume that this result holds true for larger dimensions, then Table 6 shows the approximate average running needed to locate the true pattern using both the bound and a blind exhaustive search. The times were based on using an Intel Xeon CPU 2.66 GHz, which takes about 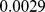 seconds to compute the bound for one pattern. In the exhaustive search it is assumed that the true model will be found after checking half of the models on the average.

**Table 6:** Approximation of the average running time before finding the true pattern.

	1000 SNPs		10,000 SNPs		100,000 SNPs	
	Bound	Exhaustive	Bound	Exhaustive	Bound	Exhaustive
2-SNP	10 sec	12 min	16 min	20 hours	28 hours	84 days
3-SNP	7 min	3 days	5 days	8 years	14 years	7,662 years
4-SNP	1 day	2 years	14 years	19,146 years	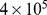 years	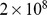 years

Notice that the bound always performs about an order of magnitude better than the exhaustive search as far as the dimension that it can handle in an acceptable amount of time. That is, in the case of the 2-SNP models the bound can handle 100,000 SNPs whereas the exhaustive search can only handle 10,000 SNPs; in the case of the 3-SNP models the bound can handle 10,000 SNPs whereas the exhaustive search can only handle 1000 SNPs; and in the case of the 4-SNP models the bound can handle 1000 SNPs whereas the exhaustive search cannot.

These results indicate that in the case of pure epistasis the bound can often locate the true pattern much sooner than would be expected by chance, and offers a substantial improvement over using the score to locate that pattern.

### Semi-Synthetic Datasets

**Figure 7:**
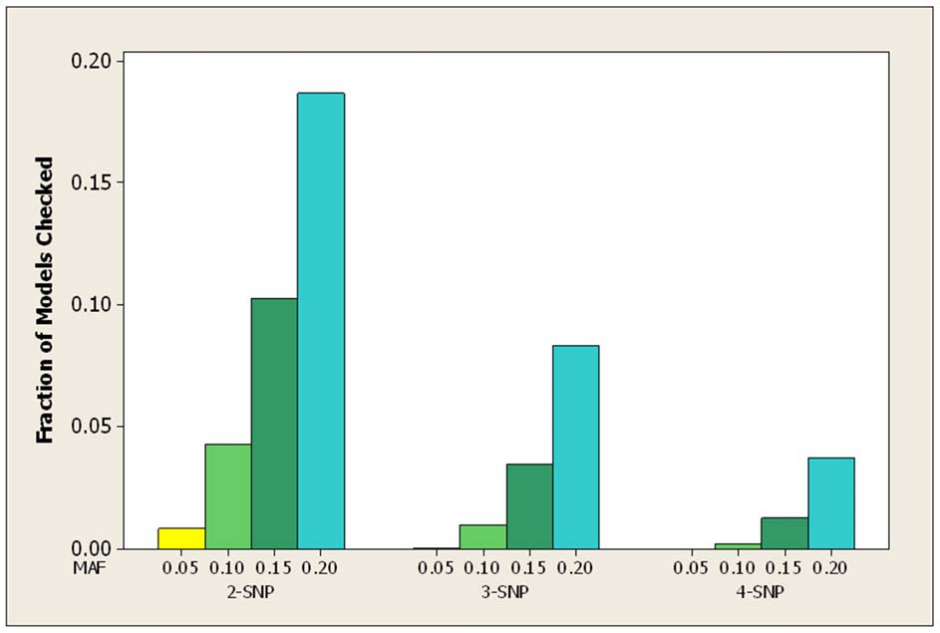
For the LOAD datasets, the average ratio of the number of patterns checked by the bound to the number of patterns checked by the score before each finds the true pattern.

**Figure 8:**
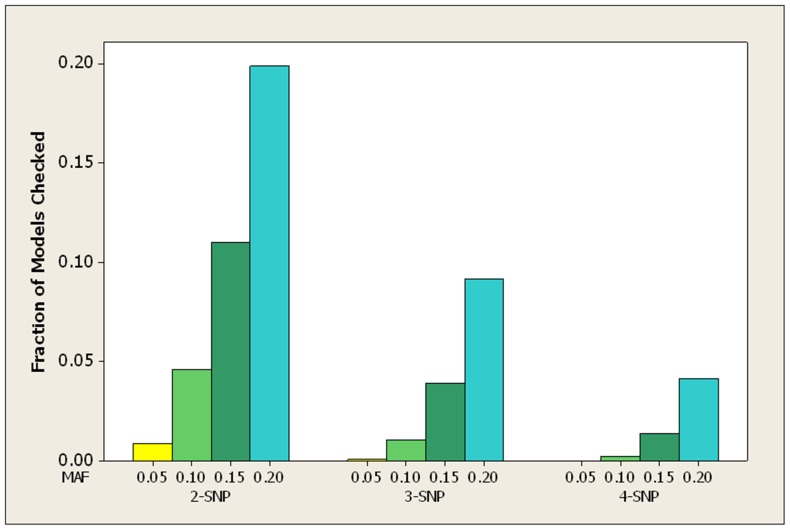
For the breast cancer datasets, the average fraction of the total number of patterns checked before the bound finds the true pattern.

For the LOAD datasets, Table 7 shows the average over all 100 datasets of the fraction of patterns checked by both the bound and the score before the true pattern was found. Figure 7 show the average fraction of patterns checked by the bound in graphical format. Table 8 and Figure 8 show the same information for the breast cancer datasets.

**Table 7:** For the LOAD datasets, average fraction over all 100 datasets of the total number of patterns checked before finding the true pattern.

MAF	2-SNP		3-SNP		4-SNP	
	Bound	Score	Bound	Score	Bound	Score
0.05	0.008	0.054	0.0003	0.017	0.0001	0.006
0.1	0.043	0.170	0.009	0.085	0.002	0.044
0.15	0.102	0.274	0.035	0.206	0.012	0.124
0.2	0.187	0.393	0.083	0.353	0.037	0.281

**Table 8:** For the breast cancer datasets, average fraction over all 100 datasets of the total number of patterns checked before finding the true pattern.

MAF	2-SNP		3-SNP		4-SNP	
	Bound	Score	Bound	Score	Bound	Score
0.05	0.008	0.045	0.0008	0.014	0.00009	0.004
0.1	0.045	0.137	0.010	0.079	0.002	0.045
0.15	0.109	0.272	0.038	0.173	0.013	0.120
0.2	0.199	0.370	0.092	0.283	0.041	0.258

The results are similar to those for the simulated datasets. In the case of both the LOAD and the breast cancer datasets, when the MAF is small (0.05 or 0.1) the bound never checks more than 5% of the patterns before finding the pattern representing the interacting SNPs, and it always checks substantially fewer patterns than the score.

Consider the following example. In the case of the LOAD dataset, there are 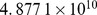 2-SNP patterns. When the MAF is 0.05, the bound checks only 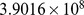 of these patterns. Using an Intel Xeon CPU 2.66 GHz, it takes about 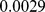 seconds to compute the score or the bound for one pattern. So an exhaustive could be expected to find the true pattern in about 2.24 years. However, the bound can be expected to find the true pattern in about 13.09 days.

It is well known that the APOE gene is associated with LOAD [12], [13]. The APOE gene has three common variants 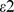, 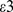 and 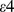. The least risk is associated with the 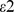 allele, while each copy of the 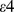 allele increases risk. As mentioned above, Reiman et al. [13] developed a GWAS late onset Alzheimer's disease (LOAD) dataset on 312,317 SNPs and APOE status. These researchers investigated pairs of loci, where one locus is APOE status, in order to possibly learn epistatic interactions of other loci with APOE. They performed separate analyses in APOE 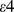 carrier and APOE 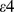 non-carriers. They found that 10 of the 25 SNPs with the most significant LOAD association in APOE 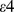 were located in the GAB2 gene. They did not find that the *GAB2* gene is significantly associated with LOAD in the *APOE* 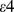 non-carriers. Reiman et al. [13] provide biological evidence that GAB2 modifies LOAD risk in APOE є4 carriers.

Using this same dataset, Jiang et al. [37] scored all 2-SNP patterns where one of the loci is the APOE gene and the other loci is one of the 312, 317 SNPs being investigated. Eight of the ten highest scoring patterns contained a GAB2 SNP, substantiating the results in [13].

This discovery of a possible interaction between GAB2 and APOE was achieved because of APOE's high marginal effect. Table 9 compares this sort of result to our result concerning the discovery of the strict epistatic interactions we injected into the LOAD dataset. The average score and bound over all SNPs were −950.46 and −17.898 respectively. We see from Table 9 that APOE has an extremely high score (by far higher than any other 1-SNP pattern), and that the GAB2 SNPs have both unremarkable scores and bounds. However, due to APOE's high score, it was reasonable and computationally efficient to investigate the interaction of every SNP with APOE. By so doing, it was discovered that patterns including APOE and GAB2 were quite likely. Our injected SNPs have unremarkable scores. Little discovery would be expected by looking at their scores alone. However, the bounds for the SNPs are quite high (almost as high as APOE's bound) when the MAF is 0.05, and they decrease as the MAF increases. This result is consistent with the results shown in Table 7. So the bounds enabled us to discover the strict epistatic interactions.

**Table 9:** The 1-SNP score, 2-SNP bound and 2-SNP score for real loci and injected loci.

Locus	1-SNP Score	2-SNP Bound	2-SNP score
APOE	−836.08	−13.74	–
rs1007837 (GAB2)	−947.88	−17.50	−831.33
rs7101429 (GAB2)	−947.16	−17.53	−830.58
rs901104 (GAB2)	−947.69	−17.57	−830.69
rs4291702 (GAB2)	−947.36	−17.66	−830.51
rs4945261 (GAB2)	−947.83	−17.59	−831.68
rs7115850 (GAB2)	−945.19	−17.89	−827.261
rs10793294 (GAB2)	−947.24	−18.39	−830.84
rs2450130 (GAB2)	−948.28	−17.60	−830.49
S1 (0.05)	−949.25	−14.60	−836.31
S2 (0.05)	−949.12	−14.42	−836.31
S1 (0.1)	−964.67	−16.42	−761.12
S2 (0.1)	−964.50	−16.24	−761.12
S1 (0.15)	−950.29	−17.56	−668.48
S2 (0.15)	−950.16	−17.54	−668.48
S1 (0.20)	−950.56	−18.42	−612.31
S2 (0.20)	−950.36	−18.60	−612.31

The 2-SNP scores for the real loci are the scores of the patterns in which the other locus is APOE. The 2-SNP scores for the injected loci are the scores of the injected patterns. For the injected SNPs, the MAFs are shown in parentheses.

It might seem odd that the average bounds for the interacting SNPs are around −18 when the MAF is 0.2 (see Table 9), which is worse than the average bound, but yet we find the pattern containing those SNPs after investigating only about 0.187 fraction of the 2-SNP patterns (see Table 7). The explanation for this is that both SNPs' bounds ordinarily ranked around the mid-point of the sorted list of all bounds. So Algorithm 1 would find the pattern containing them fairly early. The following example illustrates why this is the case. If there were 7 SNPs, there would be 21 patterns, and if the SNPs ranked as the 3^rd^ and 4^th^ SNPs, Algorithm 1 would discover the pattern containing them after investigating only 6 patterns.

Finally, note that the bound is a very loose bound. Therefore, it would not be useful in a best-search first algorithm that prunes SNPs based on their bounds and which is able to guarantee that we discovered the highest scoring pattern. However, as we have seen, the bound can be quite effective for guiding a heuristic search for high scoring patterns. In general, when we are searching for possible epistatic interactions, our concern is with finding likely patterns that we can then further investigate for biological plausibility. It is not necessary that we know that a discovered interaction has the highest score of all patterns.

## Discussion

We identified a bound on the Bayesian score of any SNP pattern that could be obtained by expanding a given SNP pattern. Using simulated datasets based on models of strict epistasis, we showed that the bound can locate the true pattern, when searching moderate-dimensional datasets, much faster than can be expected by chance. Using semi-synthetic datasets based on models of strict epistasis, we showed that the bound can locate the true pattern, when searching high-dimensional GWAS datasets, much faster than can be expected by chance. The average fraction of patterns checked before finding the true pattern was as little as 0.0004. These results indicate that the bound can be an extremely useful tool in algorithms that search high-dimensional datasets for strict epistatic interactions.

We used an algorithm that sorts the SNPs by their bounds and by their scores to test the effectiveness of the bound. Although this was an effective way to compare the bound to the score, in practice it may not be the most effective way to use the bound in a heuristic search. First, the algorithm assumes we know the number of interacting SNPs up front. Second, if, for example, we were looking for a 4-SNP interaction, we would be able to visit very few different SNPs. We plan to incorporate the bound into other algorithms we have developed such as MBS [32]. Our final algorithm will use both the bound and the score to guide our search.
